# The activation efficiency of mechanophores can be modulated by adjacent polymer composition†

**DOI:** 10.1039/d0ra09834e

**Published:** 2021-02-12

**Authors:** Sourabh Kumar, Tim Stauch

**Affiliations:** University of Bremen, Institute for Physical and Theoretical Chemistry Leobener Straße NW2 D-28359 Bremen Germany tstauch@uni-bremen.de; Bremen Center for Computational Materials Science, University of Bremen Am Fallturm 1 D-28359 Bremen Germany; MAPEX Center for Materials and Processes, University of Bremen Bibliothekstraße 1 D-28359 Bremen Germany

## Abstract

The activation efficiency of mechanophores in stress-responsive polymers is generally limited by the competing process of unspecific scission in other parts of the polymer chain. Here it is shown that the linker between the mechanophore and the polymer backbone determines the force required to activate the mechanophore. Using quantum chemical methods, it is demonstrated that the activation forces of three mechanophores (Dewar benzene, benzocyclobutene and *gem*-dichlorocyclopropane) can be adjusted over a range of almost 300% by modifying the chemical composition of the linker. The results are discussed in terms of changes in electron density, strain distribution and structural parameters during the rupture process. Using these findings it is straightforward to either significantly enhance or reduce the activation rate of mechanophores in stress-responsive materials, depending on the desired use case. The methodology is applied to switch a one-step “gating” of a mechanochemical transformation to a two-step process.

## Abstract 2

Chemical modifications of the linking units between a mechanophore and the polymer backbone can significantly enhance or reduce the rupture force of the mechanophore.

## Graphical abstract

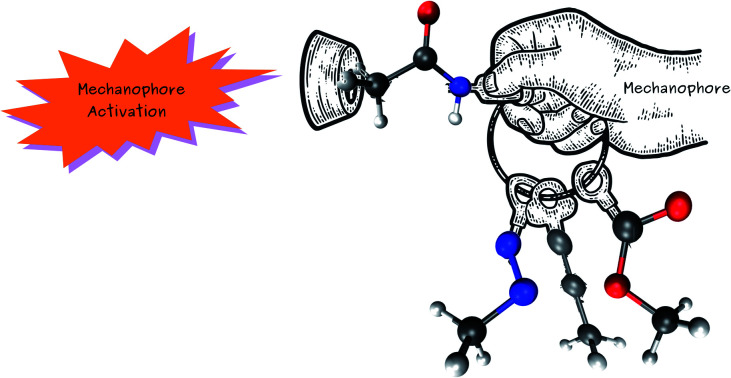

The interest in polymer mechanochemistry has been increasing steadily throughout the past two decades,^1–4^ which is due to fascinating applications of the field, such as force-induced activation of latent catalysts,^5^ the development of self-healing polymers,^6^ and optical sensing of stress and strain using mechanochromic materials.^7^ Many of these applications are enabled by mechanophores, which are small molecular subunits embedded in the polymer that respond to external forces *via* significant changes in their geometries. Experimentally, mechanophores in stress-responsive polymers can be activated by single-molecule force spectroscopy,^8^ sonochemistry,^9^ nozzle flow setups^10^ or direct mechanical manipulation.^11^ However, in many cases the activation efficiency of mechanophores in stress-responsive materials is limited and bond rupture in other parts of the polymer backbone is predominant,^12^ thus limiting the efficiency of such functional materials. On the contrary, in other application scenarios, it would be desirable to strengthen the polymer and prevent mechanophore activation below an adjustable threshold force. Therefore, a better control over the force required to activate mechanophores is highly desirable.

In computational investigations of the activation of mechanophores embedded in polymer backbones it was found that the pulling vector as well as local distortions in the linking units connecting the mechanophore to the rest of the polymer backbone (the “linkers”) have an influence on the activation efficiency of the mechanophores.^13–16^ The chemical composition of these linkers, on the other hand, has not been considered in detail in computational studies on polymer mechanochemistry, since, for simplicity, the immediate vicinity of a mechanophore is often modeled as an alkyl chain.^12^ While, experimentally, the chemical composition of the linkers is usually dictated by the synthetic route, it was shown that a stiffer polymer chain with a higher *T*_g_ transmits mechanical load more efficiently to the mechanophore.^2,5^ However, a comprehensive understanding of the influence of the chemical composition of the linkers between the polymer chain and the mechanophore on the activation of the latter remains elusive.

Using quantum mechanochemical methods,^12^ we here provide such an understanding by demonstrating that the activation force of three different mechanophores, *i.e.* Dewar benzene,^17^ benzocyclobutene^15^ and *gem*-dichlorocyclopropane,^14^ can be adjusted over a range of almost 300% by modifying the linkers that connect the mechanophore to the rest of the polymer backbone. The investigated linkers are chemically diverse and include saturated and unsaturated alkyl chains, amides, esters, an ether, a secondary amine, an imine and an azo group (Fig. 1). We use the External Force is Explicitly Included (EFEI) approach,^18–20^ which allows quantum chemical geometry optimizations under constant external stretching forces, as implemented in the Q-Chem 5.2.1 program package^21^ and apply Density Functional Theory (DFT)^22,23^ at the PBE^24^/cc-pVDZ^25^ level of theory throughout. Further computational details can be found in the ESI.†

**Fig. 1:**
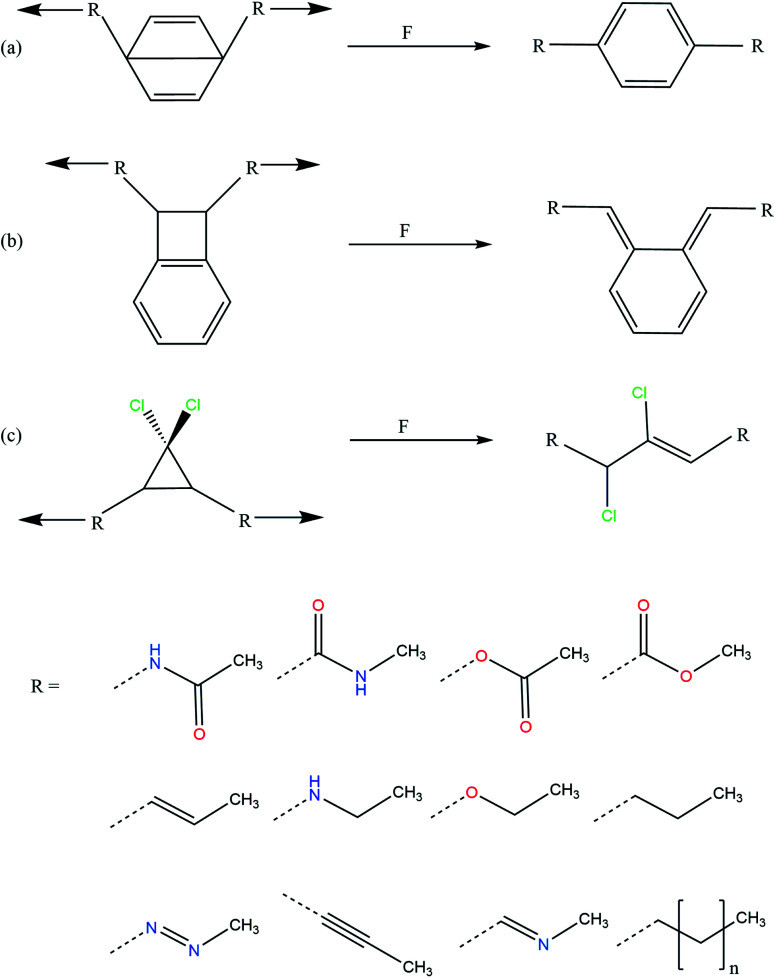
Mechanical activation pathways of the investigated mechanophores (a: Dewar benzene, b: benzocyclobutene, c: *gem*-dichlorocyclopropane) and investigated linkers.

By comparing alkyl linkers of different lengths for the three investigated mechanophores (*cf.* the ESI, Fig. S1†), it becomes apparent that the forces required to activate the mechanophores generally show an odd–even behavior with the chain length, which has been described before.^15^ Moreover, in a three-membered alkyl chain the rupture force is close to the converged value at higher chain lengths. Hence, in this study the focus lies on three-membered chains that model the polymer linkers.

To investigate the influence of linker composition on mechanophore activation, stretching forces were applied to the terminal carbon atoms of the linkers attached to each mechanophore. The calculated rupture forces for the three mechanophores connected with the investigated linkers are given in Fig. 2 (*cf.* also Table S1†). Although the three tested mechanophores are chemically diverse and display completely different activation mechanisms, the order of linkers when organizing them according to the rupture forces is similar: the lowest rupture forces are yielded by the linker that involves an alkynyl group as well as the amide linker in which the nitrogen atom is attached to the mechanophore. In *gem*-dichlorocyclopropane, for example, the amide and the alkynyl linkers lead to activation forces of only 1.35 nN and 1.64 nN, respectively, which is significantly lower than the value for the saturated alkyl linker (3.27 nN). This finding demonstrates that the computational modeling of the activation of a mechanophore in which the stretching force is transduced *via* the simplest alkyl chain yields rather high rupture forces, which might contribute to the well-known overestimation of experimental rupture forces by quantum chemistry.^12^ Experimentally, if the aim is to maximize the activation rate of mechanophores in polymers, it is recommended to place a carbon–carbon triple bond or an amide group directly next to the mechanophore.

**Fig. 2:**
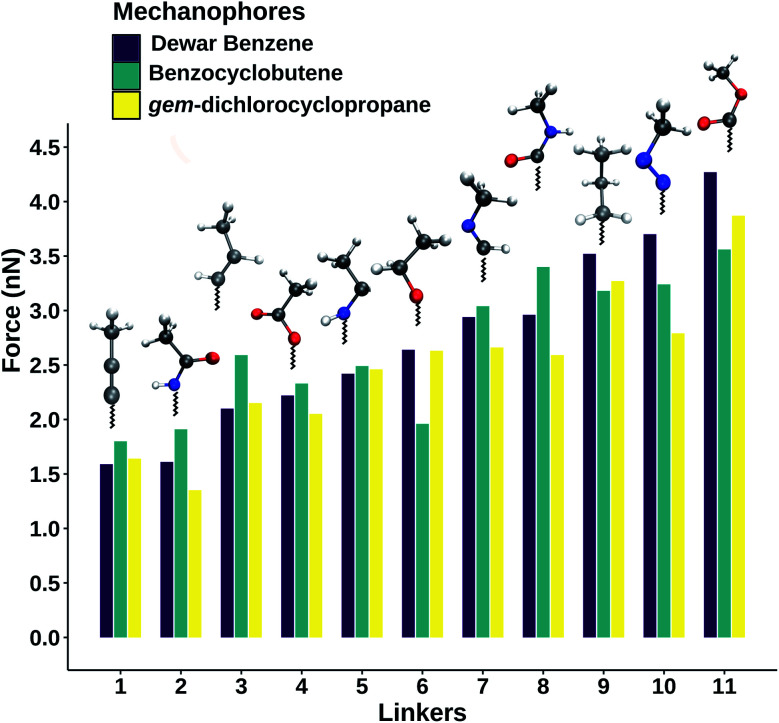
Rupture forces for the mechanical activation of Dewar benzene, benzocyclobutene and *gem*-dichlorocyclopropane using different linkers that connect the mechanophores to the rest of the polymer backbone. Color code: gray: carbon; red: oxygen; blue: nitrogen; white: hydrogen.

Linkers that yield a high rupture force include the ester and the amide that are attached to the mechanophore *via* their carbonyl groups as well as the azo linker. Connecting these species to the mechanophores in experiments would make the polymer mechanically more resilient. All in all, Fig. 2 provides a toolbox for fine-tuning the activation force of mechanophores in polymers, depending on the desired use case, simply by changing the chemical composition of the immediate vicinity of the mechanophore. The wide functionality of mechanophores (*e.g.* force-induced color-changes, fluorescence or luminescence or the release of small-molecules) would be retained.

Interestingly, using different linkers on each side of the molecule leads to intermediate rupture forces, as suggested by chemical intuition (*cf.* ESI, Table S2†): taking Dewar benzene as an example, the use of the linker that yields the lowest rupture force (the alkynyl linker) on one side of the molecule and the one yielding the highest rupture force (the ester) leads to a rupture force of 2.65 nN, which is in the middle of the region spanned by the explored space of linkers. Changing one of the linkers further allows a fine-tuning of the rupture force, which is consistent among the tested mechanophores. The data presented in Table S2† therefore suggests that application of different linkers on each side of the molecule leads to a “mixing” of their mechanical properties.

To elucidate the reason for the significant differences in rupture forces when varying the linkers, we applied the Judgement of Energy DIstribution (JEDI) analysis,^26–28^ which is a quantum chemical tool for the analysis of strain distribution in distorted molecules. However, the strain energies stored by the scissile bonds in the stretched mechanophores do not follow an easily interpretable trend that would explain the observed differences in rupture forces (*cf.* ESI, Tables S3–S5†). Instead, the distribution of strain energy is complex, signifying a more complicated rupture mechanism. Similarly, the electron densities at the bond critical points of the scissile bonds of the mechanophores, calculated with the Quantum Theory of Atoms In Molecules (QTAIM)^29^ approach, do not contribute to our understanding of the observed discrepancies in the activation forces (*cf.* ESI, Fig. S2†). While the electron densities generally decrease with increasing force, hinting towards a weakening of these bonds, these trends are similar for all mechanophores and linkers and the absolute values of the electron densities do not correlate with the rupture order.

Instead, it was found that structural parameters in the linker determine the force required for mechanophore activation. Taking Dewar benzene as an example (Fig. 3), the angle between the scissile transannular carbon–carbon bond and the first atom in the linker (*β*_0_), the angle between the mechanophore and the first two atoms in the linker (*β*_1_) and the angle between the three atoms in the chain of the linker (*β*_2_) are considered. Upon application of end-to-end stretching forces, *β*_0_ and *β*_2_ exhibit a steady increase in the case of most linkers. Conversely, the initial and final angles of *β*_1_ are almost identical in most cases, signifying that *β*_1_ is a rather stiff bond angle. A notable exception is the alkynyl linker, in which *β*_1_ initially displays a sharp decrease. Upon close inspection it becomes apparent that the initial value of *β*_1_ in a specific linker is closely related to the position of this linker in Fig. 2, with higher *β*_1_ corresponding to lower rupture force. Hence, despite the stiffness of *β*_1_, this angle plays an important role in transmitting the mechanical load to the mechanophore, and that this transmission proceeds more efficiently the more linear *β*_1_ is. Analogous results were found for benzocyclobutene and *gem*-dichlorocyclopropane (*cf.* ESI, Fig. S4, S5 and Tables S8–S11†), hinting at a general validity of these results. In a previous investigation that focused on an alkyl chain connected to benzocyclobutene,^15^ out-of-plane distortions were found to influence mechanophore activation and the role of various bond angles was discussed. Thus, the findings presented here contribute to our understanding of force transduction through polymer chains from a structural point of view.

**Fig. 3:**
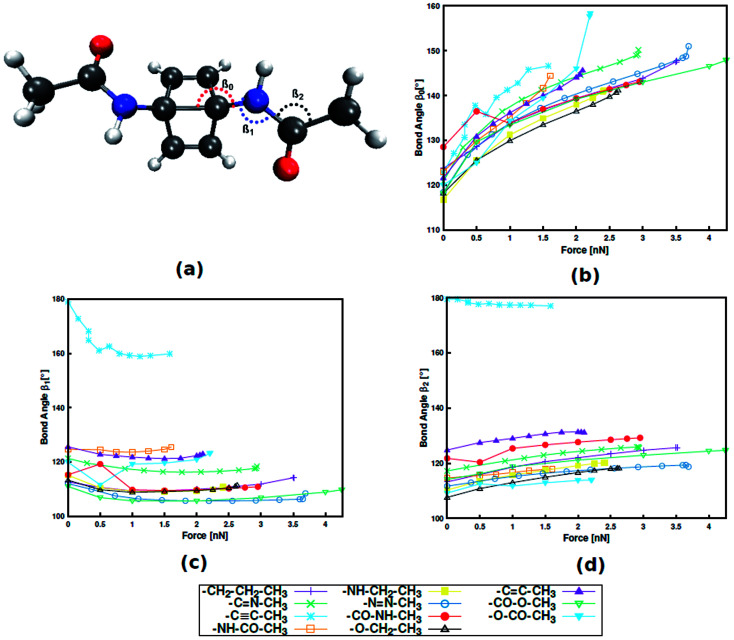
Dewar benzene with an amide linker with bond angle nomenclature (a). Progression of bond angles *β*_0_ (b), *β*_1_ (c) and *β*_2_ (d) with external stretching force.

The important role of the angle *β*_1_ is emphasized by end-to-end stretching simulations of the three mechanophores with the propyl linker in which *β*_1_ is simultaneously constrained to different values (Fig. 4). In these calculations, higher values of *β*_1_ lead to lower rupture forces, which is in accordance with the aforementioned notion that a more linear angle *β*_1_ leads to a lower rupture force. Increasing *β*_1_ to 160°, for example, leads to a reduction of the rupture force to almost a third of the initial value. Therefore, *β*_1_ is found to be the most critical coordinate for an efficient transmission of force to the mechanophore.

**Fig. 4:**
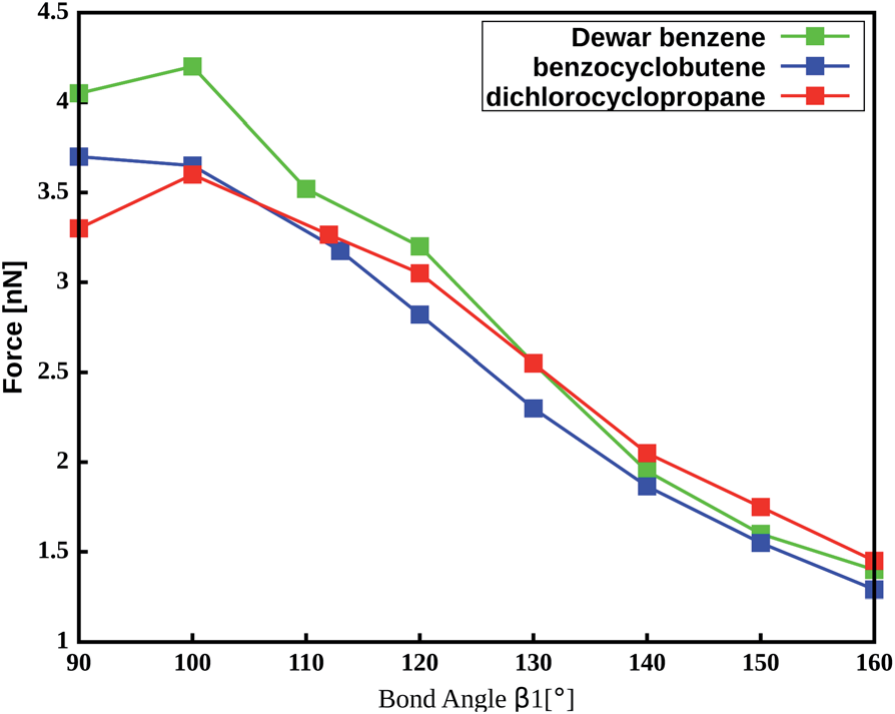
Amount of force required to activate the mechanophores using a propyl liker while constraining the angle *β*_1_.

The usefulness of the possibility to modulate the rupture forces of mechanophores by modifications of the linkers is demonstrated by considering the gating of a mechanochemical process, which has been reported recently.^30^ In the gating approach, a “weaker” mechanophore, *i.e. gem*-dichlorocyclopropane, is protected mechanically by the “stronger” cyclobutane (Fig. 5a). At the PBE/cc-pVDZ level of theory, a stretching force of 4.7 nN is required to initiate the rupture of cyclobutane, which is followed immediately by the rupture of *gem*-dichlorocyclopropane. Hence, this mechanochemical reaction can be considered as a one-step process.

**Fig. 5:**
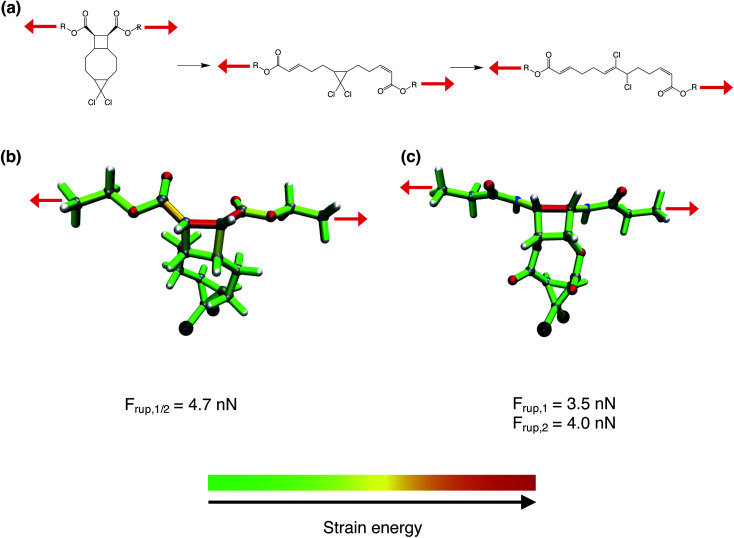
(a) Original gating approach presented in ref. 30. Red arrows signify mechanical stretching forces. (b) Distribution of strain energy^26–28^ in the bonds, bendings and torsions of a gating model system, in which both mechanophores (cyclobutane and *gem*-dichlorocyclopropane) are ruptured by a force of *F*_rup_ = 4.7 nN. (c) Distribution of strain energy in a modified system with amide linkers attached to cyclobutane (*F*_rup_ = 3.5 nN) and ester linkers attached to *gem*-dichlorocyclopropane (*F*_rup_ = 4.0 nN). Color code for the atoms: white: hydrogen; gray: carbon; red: oxygen; black: chlorine; blue: nitrogen.

We aim at a transformation of the one-step gating to a two-step process, which lends itself for incorporating a hidden length in a polymer.^31^ In the original gating approach, an ester linker is used to transmit the mechanical stretching force to cyclobutane and a simple alkyl chain connects cyclobutane to *gem*-dichlorocyclopropane (Fig. 5b). According to Fig. 2, in all investigated mechanophores the ester linker yields the highest rupture forces. By changing the linkers that transmit the stretching force to cyclobutane to amides and connecting cyclobutane to *gem*-dichlorocyclopropane *via* esters, the rupture force of cyclobutane was decreased to 3.5 nN. This force is insufficient to rupture *gem*-dichlorocyclopropane, for which 4.0 nN are required. Hence, by a simple modification of the linkers, the original one-step gating was switched to a two-step process. Since cyclobutane was not incorporated in the set of mechanophores investigated in Fig. 2, these findings lend further credibility to the general validity of the presented results.

Using the JEDI analysis,^26–28^ the reason for the reduction of rupture force in the investigated system when changing the ester linker next to cyclobutane to an amide linker are elucidated. In the original system featuring an ester linker, several bonds, bendings and torsions within the cyclobutane ring and in its vicinity are significantly strained (Fig. 5b). By contrast, the amide linker leads to an accumulation of strain almost exclusively in the scissile bond of cyclobutane (Fig. 5c), thus facilitating the rupture of this bond.

In conclusion, the calculations presented here demonstrate that the force required for the activation of a mechanophore can be tuned over a range of almost 300% simply by changing the chemical composition of the linkers between the mechanophore and the rest of the polymer chain. This paves the way for a fine-tuning of the activation rate of mechanophores in polymers when exposed to mechanical deformation or ultrasound, with the possibility to either maximize mechanophore activation or to suppress it below a threshold force. In the future, we plan to apply our findings to maximize the efficiency of flex-activation of mechanophores in polymers,^32–34^ which can be used for the release of small molecules. Moreover, it is planned to apply quantum chemical models of pressure^35–42^ to test the role of the linkers in experiments in which mechanophore activation is achieved by compression. Finally, our future studies will focus on a more realistic modeling of the polymer environment by using a multiscale model of mechanophores embedded in a polymer matrix.

## Conflicts of interest

There are no conflicts to declare.

## Supplementary Material

RA-011-D0RA09834E-s001
