# Supplementary material for: The activation efficiency of mechanophores can be modulated by adjacent polymer composition
Source: RSC Adv. 2021 Feb 12;11(13):7391–6. doi: 10.1039/d0ra09834e (PMC8695044; doi:10.1039/d0ra09834e)
Supplement: RA-011-D0RA09834E-s001 [file RA-011-D0RA09834E-s001.pdf]

Electronic Supplementary Material (ESI) for RSC Advances.
This journal is © The Royal Society of Chemistry 2021

# Supporting Information

## The Activation Efficiency of Mechanophores Can Be Modulated by Adjacent Polymer Composition

Sourabh Kumar[1] and Tim Stauch*,[1,2,3]

[1] *University of Bremen, Institute for Physical and Theoretical Chemistry, Leobener Straße NW2, D-28359 Bremen, Germany*
[2] *Bremen Center for Computational Materials Science, University of Bremen, Am Fallturm 1, D-28359 Bremen, Germany*
[3] *MAPEX Center for Materials and Processes, University of Bremen, Bibliothekstraße 1, D-28359 Bremen, Germany*

* Corresponding author. Email: tstauch@uni-bremen.de

# Contents


**1 Computational Details** **3**

**2 Dependence of Rupture Force on the Length of the Alkyl Chain** **4**

**3 Influence of the Linker on the Rupture Force** **5**

**4 Rupture Forces Using Combinations of Different Linkers** **6**

**5 JEDI Analysis** **7**

5.1 Dewar Benzene . . . . . . . . . . . . 7

5.2 Benzocyclobutene . . . . . . . . . . . . 7

5.3 *gem*-dichlorocyclopropane . . . . . . . . . . . . 8

**6 Electron Density at the Bond Critical Points** **9**

**7 Bond Angles in the Linkers** **10**

7.1 Dewar Benzene . . . . . . . . . . . . 10

7.2 Benzocyclobutene . . . . . . . . . . . . 12

7.3 *gem*-Dichlorocyclopropane . . . . . . . . . . . . 14

# 1 Computational Details

The External Force is Explicitly Included (EFEI) [1–3] method was used to optimize the molecular geometries under a constant external stretching force. In the EFEI method, external mechanical forces are applied to a molecule during a quantum chemical geometry optimization by adding a constant to the nuclear gradient of a pair of atoms that drives these atoms apart. The geometry optimization converges when the external stretching force and the internal restoring force of the molecule cancel. Rupture forces were calculated iteratively. The COnstrained Geometries simulate External Forces (COGEF) [4,5] method was used to constrain the bond angle $\beta_1$ to different values while simultaneously stretching the molecules apart with the EFEI method. In COGEF, geometrical constraints are applied during a geometry optimization, which is equivalent to the application of mechanical stress. Both EFEI and COGEF were carried out with the Q-Chem 5.2.1 [6] program package using Density Functional Theory (DFT) [7,8] at the PBE [9]/cc-pVDZ [10] level of theory based on geometries optimized at zero force. The lack of imaginary frequencies at zero force confirmed that the obtained structures are indeed true minima. The Cartesian coordinates of all structures (the three investigated mechanophores with all tested linkers) are available online as part of the Supporting Information.

The analysis of strain distribution in the mechanically distorted molecules was achieved with the Judgement of Energy DIstribution (JEDI) [11–13] analysis. Based on the harmonic approximation, the JEDI analysis quantifies the strain energies within each bond, bending and torsion of a mechanically deformed molecule. Color-coded structures of molecules under tensile stress were created with VMD 1.9.3, [14] where the strain in each bond, bending and torsion was mapped onto the bonds.

To study the role of electron density at the bond critical points, topology analysis was carried out with the Quantum Theory of Atoms In Molecules (QTAIM) [15] approach using the Multiwfn 3.7 program package. [16] The wavefunctions required for these calculations were generated with Q-Chem.

# 2 Dependence of Rupture Force on the Length of the Alkyl Chain

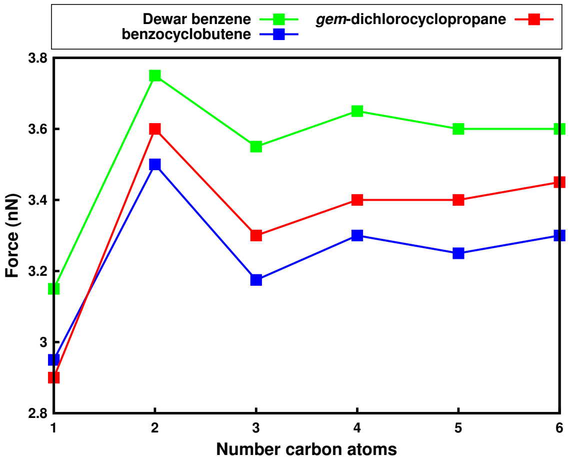


Figure S1: Amount of force required to activate the investigated mechanophores when changing the number of carbon atoms in the alkyl linker. Lines were included to guide the eye.

# 3 INFLUENCE OF THE LINKER ON THE RUPTURE FORCE

| 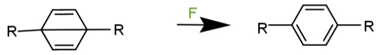 | | 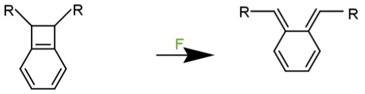 | | 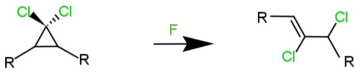 | |
|---|---|---|---|---|---|
| **Substitution** | **Rupture force (nN)** | **Substitution** | **Rupture force (nN)** | **Substitution** | **Rupture force (nN)** |
| $-C\equiv C-CH_3$ | 1.59 | $-C\equiv C-CH_3$ | 1.80 | $-NH-CO-CH_3$ | 1.35 |
| $-NH-CO-CH_3$ | 1.61 | $-NH-CO-CH_3$ | 1.91 | $-C\equiv C-CH_3$ | 1.64 |
| $-C{=}C-CH_3$ | 2.10 | $-O-CH_2-CH_3$ | 1.96 | $-O-CO-CH_3$ | 2.05 |
| $-O-CO-CH_3$ | 2.22 | $-O-CO-CH_3$ | 2.33 | $-C{=}C-CH_3$ | 2.15 |
| $-NH-CH_2-CH_3$ | 2.42 | $-NH-CH_2-CH_3$ | 2.49 | $-NH-CH_2-CH_3$ | 2.46 |
| $-O-CH_2-CH_3$ | 2.64 | $-C{=}C-CH_3$ | 2.59 | $-CO-NH-CH_3$ | 2.59 |
| $-C{=}N-CH_3$ | 2.94 | $-C{=}N-CH_3$ | 3.04 | $-O-CH_2-CH_3$ | 2.63 |
| $-CO-NH-CH_3$ | 2.96 | $-CH_2-CH_2-CH_3$ | 3.18 | $-C{=}N-CH_3$ | 2.66 |
| $-CH_2-CH_2-CH_3$ | 3.52 | $-N{=}N-CH_3$ | 3.24 | $-N{=}N-CH_3$ | 2.79 |
| $-N{=}N-CH_3$ | 3.70 | $-CO-NH-CH_3$ | 3.40 | $-CH_2-CH_2-CH_3$ | 3.27 |
| $-CO-O-CH_3$ | 4.27 | $-CO-O-CH_3$ | 3.56 | $-CO-O-CH_3$ | 3.87 |

Table S1: Rupture forces for the mechanical activation of Dewar Benzene, benzocyclobutene and *gem*-dichlorocyclopropane using different linkers that connect the mechanophores to the rest of the polymer backbone.

# 4 Rupture Forces Using Combinations of Different Linkers

| 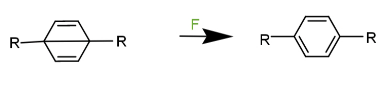 | | 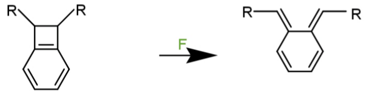 | | 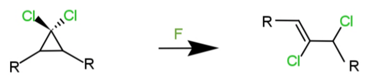 | |
|---|---|---|---|---|---|
| **Substitution (Linker1, Linker2)** | **Rupture force (nN)** | **Substitution (Linker1, Linker2)** | **Rupture force (nN)** | **Substitution (Linker1, Linker2)** | **Rupture force (nN)** |
| $-C\equiv C-CH_3$, $-O-CH_2-CH_3$ | 2.05 | $-C\equiv C-CH_3$ $-C=C-CH_3$ | 2.15 | $-NH-CO-CH_3$ $-CO-NH-CH_3$ | 1.6 |
| $-C\equiv C-CH_3$ $-CO-O-CH_3$ | 2.65 | $-C\equiv C-CH_3$ $-CO-O-CH_3$ | 2.65 | $-NH-CO-CH_3$ $-CO-O-CH_3$ | 1.9 |
| $-CO-O-CH_3$ $-O-CH_2-CH_3$ | 3.3 | $-O-CH_2-CH_3$ $-C=C-CH_3$ | 3.15 | $-CO-NH-CH_3$ $-CO-O-CH_3$ | 3.0 |

Table S2: Rupture forces for the mechanical activation of Dewar Benzene, benzocyclobutene and *gem*-dichlorocyclopropane using combinations of different linkers.

# 5 JEDI ANALYSIS

## 5.1 DEWAR BENZENE

| Substitution | Strain percentage | | | Absolute energy (a.u.) |
|---|---|---|---|---|
| | **Bond lengths** | **Bond angles** | **Dihedral angles** | |
| $-NH-CO-CH_3$ | 27.55 | 18.03 | 54.42 | 0.0134181807 |
| $-C{=}C-CH_3$ | 24.09 | 22.55 | 53.36 | 0.0109785290 |
| $-CO-O-CH_3$ | 39.17 | 20.34 | 40.49 | 0.033472350 |

Table S3: Percentage of total strain stored in the bond lengths, bond angles and dihedral angles of Dewar benzene with different linkers as well as strain energy in the scissile C−C bond at a stretching force that is 0.01 nN lower than the rupture force.

## 5.2 BENZOCYCLOBUTENE

| Substitution | Strain percentage | | | Absolute energy (a.u.) |
|---|---|---|---|---|
| | **Bond lengths** | **Bond angles** | **Dihedral angles** | |
| $-NH-CO-CH_3$ | 12.41 | 19.14 | 68.45 | 0.02943622 |
| $-C{=}C-CH_3$ | 35.09 | 27.78 | 37.13 | 0.0288327 |
| $-CO-O-CH_3$ | 21.11 | 20.24 | 58.66 | 0.0295591 |

Table S4: Percentage of total strain stored in the bond lengths, bond angles and dihedral angles of benzocyclobutene with different linkers as well as strain energy in the scissile C−C bond at a stretching force that is 0.01 nN lower than the rupture force.

## 5.3 *gem*-DICHLOROCYCLOPROPANE

| **Substitution** | **Strain percentage** | | | **Absolute energy (a.u.)** |
|---|---|---|---|---|
| | **Bond lengths** | **Bond angles** | **Dihedral angles** | |
| $-NH-CO-CH_3$ | 4.13 | 2.63 | 93.24 | 0.0050843 |
| $-C=C-CH_3$ | 22.05 | 25.90 | 52.05 | 0.008019 |
| $-CO-O-CH_3$ | 26.09 | 19.39 | 54.51 | 0.0277913 |

Table S5: Percentage of total strain stored in the bond lengths, bond angles and dihedral angles of *gem*-dichlorocyclopropane with different linkers as well as strain energy in the scissile C−C bond at a stretching force that is 0.01 nN lower than the rupture force.

# 6 ELECTRON DENSITY AT THE BOND CRITICAL POINTS

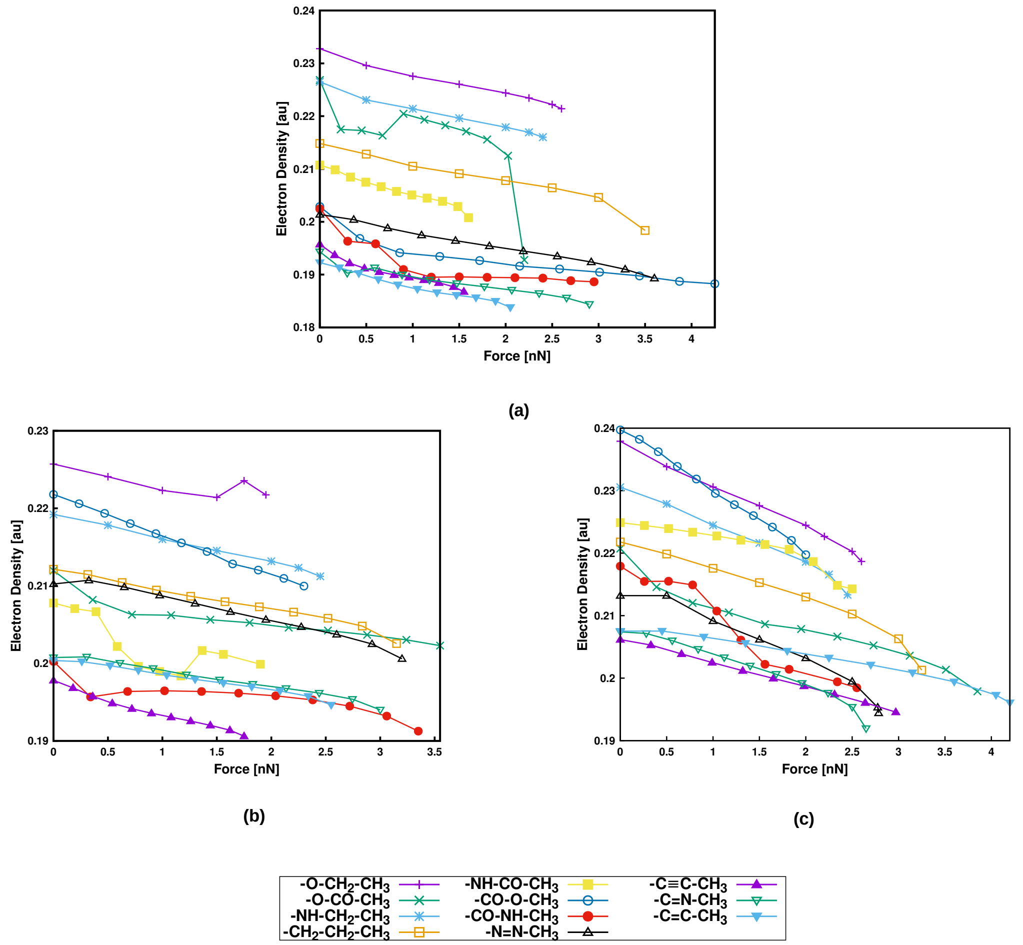


Figure S2: Electron densities in the scissile C−C bonds in Dewar benzene (a), benzocyclobutene (b) and *gem*-dichlorocyclopropane (c) using different linkers, as a function of external stretching force.

# 7 Bond Angles in the Linkers

## 7.1 Dewar Benzene

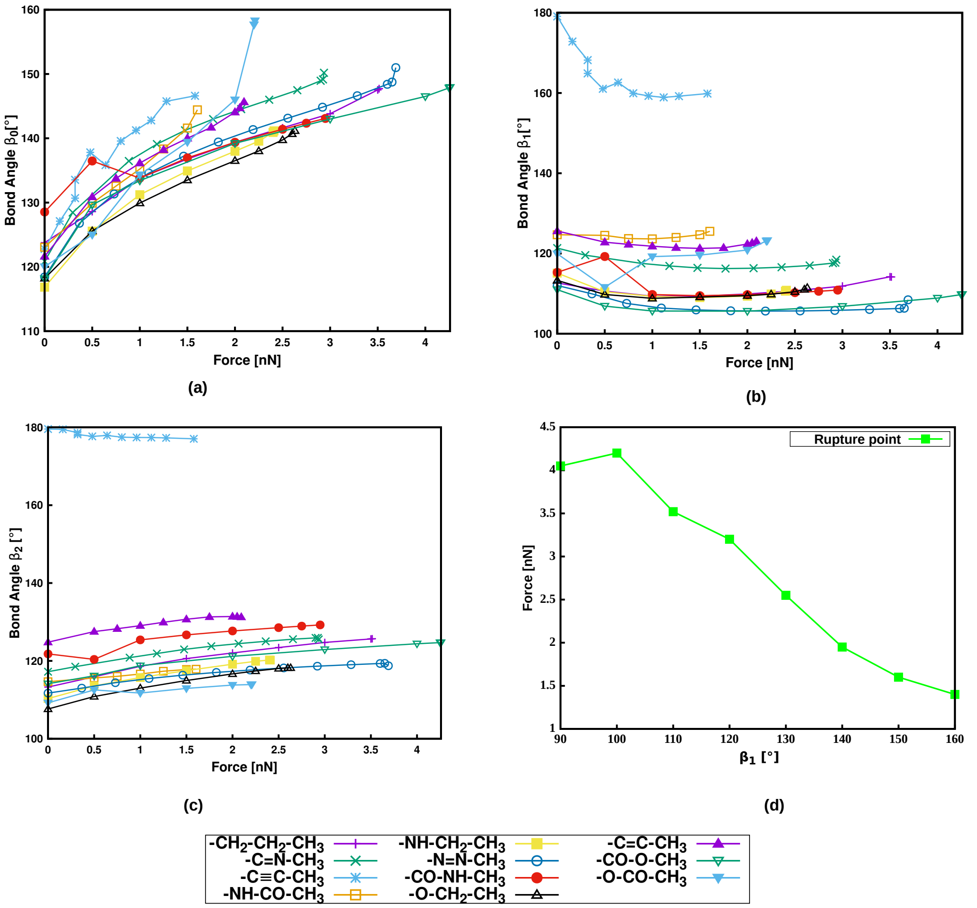


Figure S3: Bond angles $\beta_0$ (a), $\beta_1$ (b) and $\beta_2$ (c) as a function of external stretching force applied to the terminal methyl groups in Dewar benzene with different linkers. (d) Rupture forces as a function of the constrained angle $\beta_1$.

| Substitution | $\beta_0$ (initial) | $\beta_0$ (final) |
|---|---|---|
| $-C{\equiv}C-CH_3$ | 122.78 | 146.61 |
| $-NH-CO-CH_3$ | 123.05 | 144.43 |
| $-C{=}C-CH_3$ | 121.63 | 145.65 |
| $-O-CO-CH_3$ | 120.03 | 158.29 |
| $-NH-CH_2-CH_3$ | 116.81 | 141.15 |
| $-O-CH_2-CH_3$ | 118.22 | 141.17 |
| $-C{=}N-CH_3$ | 120.86 | 150.19 |
| $-CO-NH-CH_3$ | 128.53 | 143.09 |
| $-CH_2-CH_2-CH_3$ | 123.76 | 147.72 |
| $-N{=}N-CH_3$ | 118.43 | 150.99 |
| $-CO-O-CH_3$ | 118.18 | 147.93 |

Table S6: Initial (F = 0) and final (0.01 nN before bond rupture) values of the bond angle $\beta_0$ in Dewar benzene.

| Substitution | $\beta_1$ (initial) | $\beta_2$ (initial) | $\beta_1$ (final) | $\beta_2$ (final) |
|---|---|---|---|---|
| $-C{\equiv}C-CH_3$ | 179.08 | 179.64 | 159.86 | 177.05 |
| $-NH-CO-CH_3$ | 124.67 | 114.65 | 125.53 | 117.87 |
| $-C{=}C-CH_3$ | 125.61 | 124.81 | 123.05 | 131.26 |
| $-O-CO-CH_3$ | 119.94 | 109.22 | 123.24 | 113.95 |
| $-NH-CH_2-CH_3$ | 115.12 | 110.32 | 110.83 | 120.20 |
| $-O-CH_2-CH_3$ | 113.29 | 107.64 | 111.44 | 118.20 |
| $-C{=}N-CH_3$ | 121.34 | 117.22 | 118.44 | 125.51 |
| $-CO-NH-CH_3$ | 115.54 | 122.68 | 110.92 | 129.95 |
| $-CH_2-CH_2-CH_3$ | 112.67 | 113.27 | 114.15 | 125.69 |
| $-N{=}N-CH_3$ | 112.03 | 111.72 | 108.46 | 118.75 |
| $-CO-O-CH_3$ | 110.98 | 114.14 | 109.80 | 124.70 |

Table S7: Initial (F = 0) and final (0.01 nN before bond rupture) values of the bond angles $\beta_1$ and $\beta_2$ in Dewar benzene.

## 7.2 BENZOCYCLOBUTENE

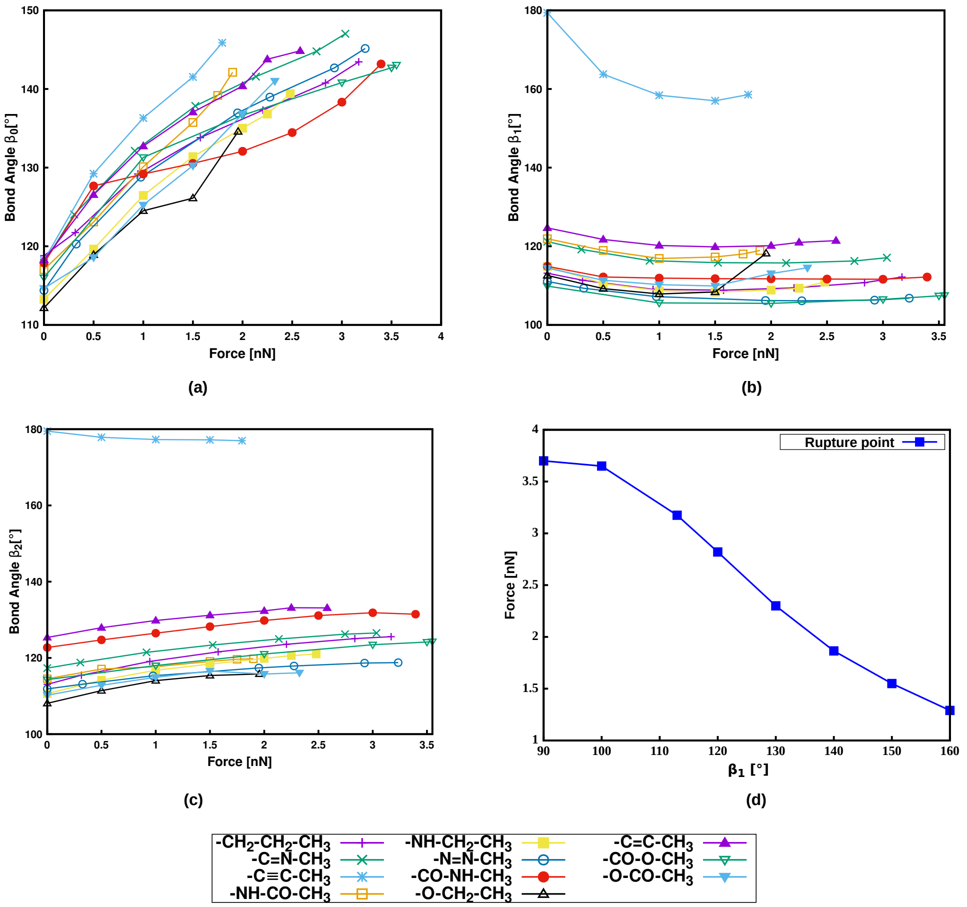


Figure S4: Bond angles $\beta_0$ (a), $\beta_1$ (b) and $\beta_2$ (c) as a function of external stretching force applied to the terminal methyl groups in benzocyclobutene with different linkers. (d) Rupture forces as a function of the constrained angle $\beta_1$.

| Substitution | $\beta_0$ (initial) | $\beta_0$ (final) |
|---|---|---|
| $-C{\equiv}C-CH_3$ | 118.171 | 145.894 |
| $-NH-CO-CH_3$ | 120.048 | 140.658 |
| $-O-CH_2-CH_3$ | 112.153 | 134.608 |
| $-O-CO-CH_3$ | 113.25 | 141.284 |
| $-NH-CH_2-CH_3$ | 111-734 | 138.678 |
| $-C{=}C-CH_3$ | 118.263 | 144.854 |
| $-C{=}N-CH_3$ | 117.916 | 147.029 |
| $-CH_2-CH_2-CH_3$ | 118.802 | 143.453 |
| $-N{=}N-CH_3$ | 114.382 | 145.159 |
| $-CO-NH-CH_3$ | 117.876 | 143.183 |
| $-CO-O-CH_3$ | 119.926 | 142.93 |

Table S8: Initial (F = 0) and final (0.01 nN before bond rupture) values of the bond angle $\beta_0$ in benzocyclobutene.

| Substitution | $\beta_1$ (initial) | $\beta_2$ (initial) | $\beta_1$ (final) | $\beta_2$ (final) |
|---|---|---|---|---|
| $-C{\equiv}C-CH_3$ | 179.49 | 179.31 | 158.53 | 176.95 |
| $-NH-CO-CH_3$ | 125.71 | 115.08 | 126.032 | 118.005 |
| $-O-CH_2-CH_3$ | 112.59 | 108.08 | 118.19 | 115.79 |
| $-O-CO-CH_3$ | 115.81 | 109.73 | 114.55 | 115.99 |
| $-NH-CH_2-CH_3$ | 113.85 | 110.82 | 109.04 | 120.93 |
| $-C{=}C-CH_3$ | 124.68 | 125.36 | 121.42 | 133.12 |
| $-C{=}N-CH_3$ | 121.175 | 117.33 | 117.089 | 126.53 |
| $-CH_2-CH_2-CH_3$ | 113.14 | 113.07 | 112.17 | 125:57 |
| $-N{=}N-CH_3$ | 111.05 | 111.87 | 106.79 | 118.77 |
| $-CO-NH-CH_3$ | 114.89 | 122.72 | 112.17 | 131.47 |
| $-CO-O-CH_3$ | 109.87 | 114.30 | 107.51 | 124.29 |

Table S9: Initial (F = 0) and final (0.01 nN before bond rupture) values of the bond angles $\beta_1$ and $\beta_2$ in benzocyclobutene.

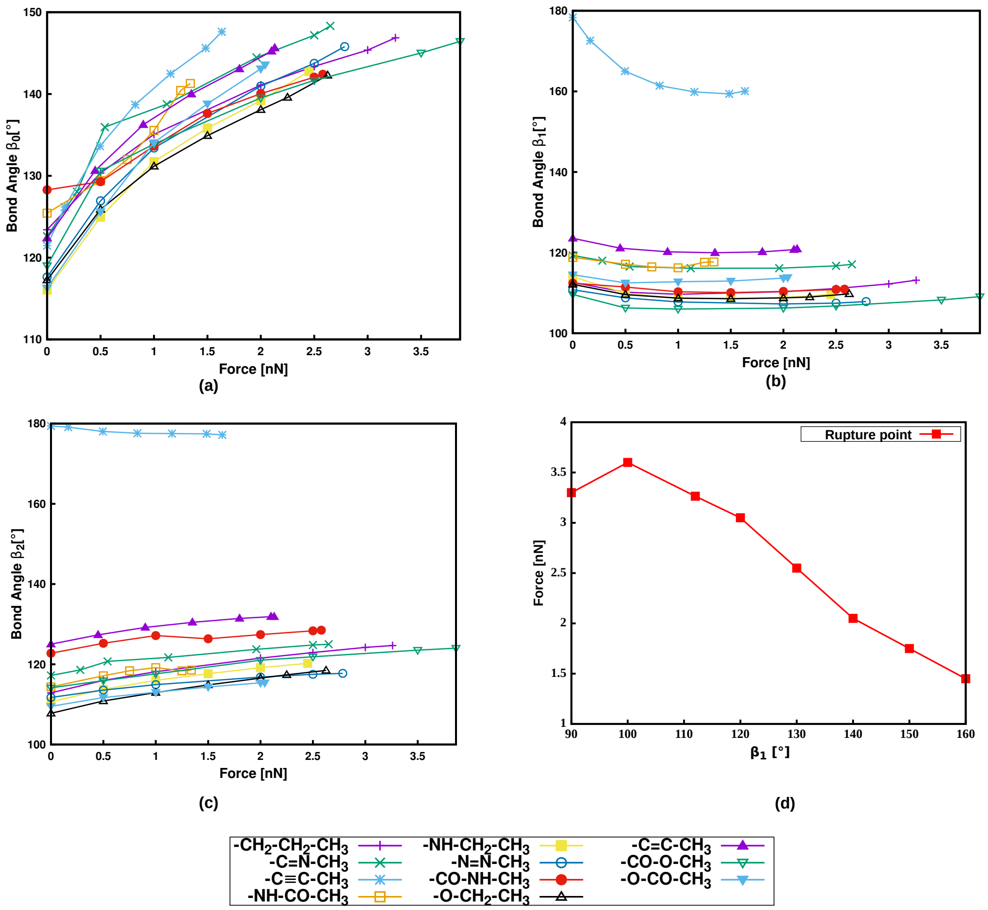


Figure S5: Bond angles $\beta_0$ (a), $\beta_1$ (b) and $\beta_2$ (c) as a function of external stretching force applied to the terminal methyl groups in *gem*-dichlorocyclopropane with different linkers. (d) Rupture forces as a function of the constrained angle $\beta_1$.

| **Substitution** | $\beta_0$ **(initial)** | $\beta_0$ **(final)** |
|---|---|---|
| $-NH-CO-CH_3$ | 129.373 | 141.233 |
| $-C{\equiv}C-CH_3$ | 121.469 | 147.619 |
| $-O-CO-CH_3$ | 116.307 | 143.588 |
| $-C{=}C-CH_3$ | 122.318 | 145-621 |
| $-NH-CH_2-CH_3$ | 116.030 | 142:323 |
| $-CO-NH-CH_3$ | 128.291 | 142.284 |
| $-O-CH_2-CH_3$ | 117.126 | 142.300 |
| $-C{=}N-CH_3$ | 122.61 | 148.311 |
| $-N{=}N-CH_3$ | 117.639 | 141.101 |
| $-CH_2-CH_2-CH_3$ | 123.419 | 146.877 |
| $-CO-O-CH_3$ | 119.029 | 146.292 |

Table S10: Initial (F = 0) and final (0.01 nN before bond rupture) values of the bond angle $\beta_0$ in *gem*-dichlorocyclopropane.

| **Substitution** | $\beta_1$ **(initial)** | $\beta_2$ **(initial)** | $\beta_1$ **(final)** | $\beta_2$ **(final)** |
|---|---|---|---|---|
| $-NH-CO-CH_3$ | 118.82 | 114.37 | 117.70 | 118.56 |
| $-C{\equiv}C-CH_3$ | 178.33 | 179.39 | 164.78 | 178.10 |
| $-O-CO-CH_3$ | 114.53 | 109.81 | 113.84 | 115.41 |
| $-C{=}C-CH_3$ | 123.63 | 124-91 | 120.89 | 131.85 |
| $-NH-CH_2-CH_3$ | 114.45 | 110.29 | 111.79 | 120.37 |
| $-CO-NH-CH_3$ | 116.98 | 121.20 | 110.97 | 128.45 |
| $-O-CH_2-CH_3$ | 112.14 | 107.78 | 109.76 | 118.48 |
| $-C{=}N-CH_3$ | 119.31 | 117-22 | 117.10 | 124.97 |
| $-N{=}N-CH_3$ | 110.79 | 111-74 | 107.94 | 117.66 |
| $-CH_2-CH_2-CH_3$ | 112.66 | 112-88 | 113.17 | 124.67 |
| $-CO-O-CH_3$ | 108.43 | 114.10 | 109.08 | 124.09 |

Table S11: Initial (F = 0) and final (0.01 nN before bond rupture) values of the bond angles $\beta_1$ and $\beta_2$ in *gem*-dichlorocyclopropane.

# References


[1] M. T. Ong, J. Leiding, H. Tao, A. M. Virshup, and T. J. Martínez, "First Principles Dynamics and Minimum Energy Pathways for Mechanochemical Ring Opening of Cyclobutene.," *J. Am Chem. Soc.*, vol. 131, pp. 6377–6379, 2009.

[2] J. Ribas-Arino, M. Shiga, and D. Marx, "Understanding Covalent Mechanochemistry.," *Angew. Chem. Int. Ed.*, vol. 48, pp. 4190–4193, 2009.

[3] K. Wolinski and J. Baker, "Theoretical predictions of enforced structural changes in molecules," *Mol. Phys.*, vol. 107, no. 22, pp. 2403–2417, 2009.

[4] M. K. Beyer, "The mechanical strength of a covalent bond calculated by density functional theory," *J. Chem. Phys.*, vol. 112, p. 7307, 2000.

[5] E. A. Nikitina, V. D. Khavryutchenko, E. F. Sheka, H. Barthel, and J. Weis, "Deformation of Poly(dimethylsiloxane) Oligomers under Uniaxial Tension: Quantum Chemical View," *J. Phys. Chem. A*, vol. 103, pp. 11355–11365, 1999.

[6] Y. Shao, Z. Gan, E. Epifanovsky, A. T. B. Gilbert, M. Wormit, J. Kussmann, A. W. Lange, A. Behn, J. Deng, X. Feng, D. Ghosh, M. Goldey, P. R. Horn, L. D. Jacobson, I. Kaliman, R. Z. Khalliulin, T. Kuś, A. Landau, J. Liu, E. I. Proynov, Y. M. Rhee, R. M. Richard, M. A. Rohrdanz, R. P. Steele, E. J. Sundstrom, H. L. Woodcock, P. M. Zimmerman, D. Zuev, B. Albrecht, E. Alguire, B. Austin, G. J. O. Beran, Y. A. Bernard, E. Berquist, K. Brandhorst, K. B. Bravaya, S. T. Brown, D. Casanova, C.-M. Chang, Y. Chen, S. H. Chien, K. D. Closser, D. L. Crittenden, M. Diedenhofen, R. A. DiStasio, H. Do, A. D. Dutoi, R. G. Edgar, S. Fatehi, L. Fusti-Molnar, A. Ghysels, A. Golubeva-Zadorozhnaya, J. Gomes, M. W. D. Hanson-Heine, P. H. P. Harbach, A. W. Hauser, E. G. Hohenstein, Z. C. Holden, T.-C. Jagau, H. Ji, B. Kaduk, K. Khistyaev, J. Kim, J. Kim, R. A. King, P. Klunzinger, D. Kosenkov, T. Kowalczyk, C. M. Krauter, K. U. Lao, A. D. Laurent, K. V. Lawler, S. V. Levchenko, C. Y. Lin, F. Liu, E. Livshits, R. C. Lochan, A. Luenser, P. Manohar, S. F. Manzer, S.-P. Mao, N. Mardirossian, A. V. Marenich, S. A. Maurer, N. J. Mayhall, E. Neuscamman, C. M. Oana, R. Olivares-Amaya, D. P. O'Neill, J. A. Parkhill, T. M. Perrine, R. Peverati, A. Prociuk, D. R. Rehn, E. Rosta, N. J. Russ, S. M. Sharada, S. Sharma, D. W. Small, A. Sodt, T. Stein, D. Stück, Y.-C. Su, A. J. W. Thom, T. Tsuchimochi, V. Vanovschi, L. Vogt, O. Vydrov, T. Wang, M. A. Watson, J. Wenzel, A. White, C. F. Williams, J. Yang, S. Yeganeh, S. R. Yost, Z.-Q. You, I. Y. Zhang, X. Zhang, Y. Zhao, B. R. Brooks, G. K. L. Chan, D. M. Chipman, C. J. Cramer, W. A. Goddard III, M. S. Gordon, W. J. Hehre, A. Klamt, H. F. Schaefer, M. W. Schmidt, C. D. Sherrill, D. G. Truhlar, A. Warshel, X. Xu, A. Aspuru-Guzik, R. Baer, A. T. Bell, N. A. Besley, J.-D. Chai, A. Dreuw, B. D. Dunietz, T. R. Furlani, S. R. Gwaltney, C.-P. Hsu, Y. Jung, J. Kong, D. S. Lambrecht, W. Liang, C. Ochsenfeld, V. A. Rassolov, L. V. Slipchenko, J. E. Subotnik, T. Van Voorhis, J. M. Herbert, A. I. Krylov, P. M. W. Gill, and M. Head-Gordon, "Advances in molecular quantum chemistry contained in the Q-Chem 4 program package," *Mol. Phys.*, vol. 113, pp. 184–215, sep 2014.

[7] P. Hohenberg and W. Kohn, "Inhomogeneous Electron Gas," *Phys. Rev.*, vol. 136, pp. 864–871, 1964.

[8] W. Kohn and L. J. Sham, “Self-Consistent Equations Including Exchange and Correlation Effects*,” *Phys. Rev.*, vol. 140, pp. 1133–1138, 1965.

[9] J. P. Perdew, K. Burke, and M. Ernzerhof, “Generalized Gradient Approximation Made Simple,” *Phys. Rev. Lett.*, vol. 77, pp. 3865–3868, 1996.

[10] T. H. Dunning, “Gaussian basis sets for use in correlated molecular calculations. I. The atoms boron through neon and hydrogen,” *J. Chem. Phys.*, vol. 90, pp. 1007–1023, 1989.

[11] T. Stauch and A. Dreuw, “A quantitative quantum-chemical analysis tool for the distribution of mechanical force in molecules,” *J. Chem. Phys.*, vol. 140, no. 13, p. 134107, 2014.

[12] T. Stauch and A. Dreuw, “On the use of different coordinate systems in mechanochemical force analyses,” *J. Chem. Phys.*, vol. 143, p. 074118, 2015.

[13] T. Stauch and A. Dreuw, “Quantum Chemical Strain Analysis For Mechanochemical Processes,” *Acc. Chem. Res.*, vol. 50, pp. 1041–1048, 2017.

[14] W. Humphrey, A. Dalke, and K. Schulten, “VMD: Visual Molecular Dynamics,” *J. Mol. Graphics*, vol. 14, pp. 33–38, 1996.

[15] R. F. W. Bader, “Atoms in Molecules,” *Acc. Chem. Res.*, vol. 18, pp. 9–15, 1985.

[16] T. Lu and F. Chen, “Multiwfn: A Multifunctional Wavefunction Analyzer,” *J. Comput. Chem.*, vol. 33, pp. 580–592, 2012.
